# Adaptation to a seasonally varying environment: a strong latitudinal cline in reproductive diapause combined with high gene flow in *Drosophila montana*

**DOI:** 10.1002/ece3.14

**Published:** 2011-10

**Authors:** Venera I Tyukmaeva, Tiina S Salminen, Maaria Kankare, K Emily Knott, Anneli Hoikkala

**Affiliations:** Department of Biological and Environmental Science, Centre of Excellence in Evolutionary ResearchP.O. Box 35, 40014 University of Jyväskylä, Finland

**Keywords:** Critical day length, gene flow, genetic variation, microsatellites, population structure, seasonal adaptation

## Abstract

Adaptation to seasonal changes in the northern hemisphere includes an ability to predict the forthcoming cold season from gradual changes in environmental cues early enough to prepare for the harsh winter conditions. The magnitude and speed of changes in these cues vary between the latitudes, which induces strong selection pressures for local adaptation.

We studied adaptation to seasonal changes in *Drosophila montana*, a northern maltfly, by defining the photoperiodic conditions leading to adult reproductive diapause along a latitudinal cline in Finland and by measuring genetic differentiation and the amount of gene flow between the sampling sites with microsatellites. Our data revealed a clear correlation between the latitude and the critical day length (CDL), in which half of the females of different cline populations enter photoperiodic reproductive diapause. There was no sign of limited gene flow between the cline populations, even though these populations showed isolation by distance. Our results show that local adaptation may occur even in the presence of high gene flow, when selection for locally adaptive life-history traits is strong. A wide range of variation in the CDLs of the fly strains within and between the cline populations may be partly due to gene flow and partly due to the opposing selection pressures for fly reproduction and overwinter survival. This variation in the timing of diapause will enhance populations’ survival over the years that differ in the severity of the winter and in the length of the warm period and may also help them respond to long-term changes in environmental conditions.

## Introduction

Local adaptation is a crucial source of biodiversity and one of the key processes that may lead to speciation. Several scenarios for sympatric and parapatric speciation suggest that this kind of adaptation plays an important role especially in the early stages of population divergence (reviewed by Schluter 2001; Via 2009), with natural selection favoring the best phenotype in a given habitat and gene flow and genetic drift diluting the effects of selection. An efficient way to study the balance between selection and gene flow is to trace phenotypic and genetic divergence of parapatric populations forming latitudinal or altitudinal clines. Clinal variation has been detected in several taxonomic groups for a variety of life-history traits associated with gradually changing environmental variables, for example, body mass, fecundity, and diapause, at distances varying from a few meters to hundreds of kilometers (reviewed by Endler 1977; Tauber and Tauber 1986; Danks 1987).

Many insect and other arthropod species living in a seasonally varying environment spend the unfavorable season in diapause, a neurohormonally mediated, dynamic developmental state of low metabolic activity (Denlinger 2002). This state should be distinguished from quiescence, which is generally regarded as a direct and immediate response to adverse environmental factors that can take place at any stage of the life cycle and be quickly reversed to normal conditions. Insect species can possess a variety of diapause strategies depending on their life cycle and ecological needs to overcome unfavorable periods, such as cold winters or dry and hot summers. These strategies are called facultative when they are determined by environmental conditions that the organism or its parents experience, and obligatory when an organism's development temporarily stops at a certain point of the life cycle regardless of environmental factors. Diapause can take place at different stages of metamorphosis from embryo to adults, depending on the species and its environment (Bale and Hayward 2010).

Diapause can be triggered by a variety of environmental cues, such as photoperiod, temperature, and/or humidity (Tauber and Tauber 1986). For a majority of organisms living in temperate zones, photoperiod is the most reliable cue evoking diapause as it changes gradually around the year regardless of other environmental cues. As the photoperiodic changes are tightly linked to latitude, they help the individuals to keep track of changing seasons and prepare for forthcoming cold periods in advance. In some species, individuals can perceive the photoperiodic cues evoking diapause a long time before the actual diapause response takes place and store this information over several developmental stages or even generations (Tauber and Tauber 1986).

In adult females, photoperiodic reproductive diapause is defined by cessation of ovarian development and reproductive activity. Correct timing of this kind of diapause has important consequences in evolutionary and ecological trade-offs between the females’ reproduction and survival. For females that emerge in early summer, entering diapause instead of developing ovaries and producing progeny during the same season poses two kinds of risks: they may deplete their energy reservoirs already during the warm period (Hahn and Denlinger 2007), and/or they may not produce any progeny if they die before the next favorable season. Conversely, if females emerging in late summer develop ovaries and produce progeny instead of entering diapause, they and/or their progeny may not survive over the winter (Musolin and Numata 2003). The day length at which half of the females of a population enter diapause and postpone their reproduction to a more favorable season is called a critical day length (CDL). This is not only a quantitative measure, but also an important population characteristic indicating the date before which it would be beneficial for females to reproduce and produce progeny and after which the females should enter diapause.

Insect species with wide geographical distributions often show fine-tuned correlations between the quality of photoperiodic cues evoking diapause and latitude. One of the first studies on variation in insect photoperiodic responses along a latitudinal cline was carried out with the knot grass moth *Acronycta rumicis* (Danilevskii 1965). This study revealed a 5 hours difference in the CDLs for pupal diapause induction between the northernmost and southernmost populations of the cline (43°–60°N). Another well-known example of clinal variation in diapause induction is a study on the pitcher plant mosquito *Wyeomyia smithii*, where the CDLs for the initiation and maintenance of larval diapause in 22 populations along a latitudinal cline in North America were found to correlate with latitude (Bradshaw 1976). Clinal variation in adult reproductive diapause has been studied in several species of the genus *Drosophila*. Lankinen (1986) found a high correlation between the latitude and the CDL for female reproductive diapause along a latitudinal cline reaching from 41°6′ to 69°0′ in *Drosophila littoralis*, the average change in CDL being 1 h and 23 min per 5° of latitude. Ichijo (1986) showed the CDL for diapause in Japanese *D. lacertosa* to decrease significantly toward lower latitudes on the island of Hokkaido (45°25′–41°25′N), while the phenomenon was not so clear on the island of Honshu (41°30′–34°52′N) located south of Hokkaido. Moreover, there was a clear disruption in the cline between the islands (Ichijo 1986). More lately, female diapause propensity has been found to vary along a latitudinal cline of *D. melanogaster* in North America (Schmidt and Paaby 2008). However, female reproductive diapause in this species can be easily interrupted and is not so clearly under photoperiodic regulation as in the northern *Drosophila* species (Tauber and Kyriacou 2001).

Despite the large number of studies carried out on insect diapause, only a few of them (e.g., Demont et al. 2008) have analyzed population structure underlying the phenotypic differences. In the present study, we have traced variation in CDLs for female reproductive diapause within and among *D. montana* populations along a latitudinal cline in Finland (61°–67°N, about 760 km) and studied whether the population samples from different parts of the cline show any sign of genetic differentiation. *Drosophila montana* females show clear photoperiodic responses and enter adult reproductive diapause when the day length decreases below the CDL (Lumme 1978), which offers a good chance to trace the balance between the local selection pressures on the seasonal timing of female reproductive diapause and the homogenizing effect of gene flow.

## Material and Methods

### Fly material

**Figure 1:**
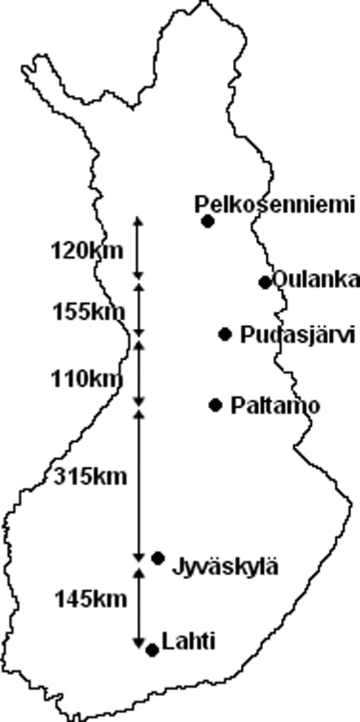
The study cline in Finland: the sampling localities and distances (km) between them.

*Drosophila montana*, a species of the *D. virilis* group, has originated in continental Asia and spread around the northern hemisphere adapting to various kinds of environmental conditions at different latitudes and altitudes (Throckmorton 1982). Our study involved flies from six localities along a 760 km long latitudinal gradient in Finland, collected in August 2008 and 2009 (Fig. 1). Information about the cline populations, photoperiodic conditions, and the codes for the isofemale lines (progeny of one wild-caught fertilized female) is given in Table 1. The lines were maintained in half-pint bottles with malt media (Lakovaara 1969) under diapause-preventing conditions (continuous light, 19°C) for about six generations before using them in the experiment. After the establishment of the lines, the founder females as well as other flies collected from the cline populations were stored in 70% ethanol for use in the population differentiation analysis.

**Table 1:** *Drosophila montana* isofemale strains used in the diapause experiment

Locality (year of collection)	Coordinates	The longest day length in nature (hours)	Isofemale line
Pelkosenniemi (2008)	67°06′N	24.00	5PTF
	27°30′E		17PTF
			4PTF
			22PTF
Oulanka (2008)	66°22′N	24.00	5OL8
	29°20′E		2OL8
			26OL8
			265OJ8
Pudasjärvi (2008)	65°21′N	22.36	6PJF
	26°59′E		9 PJF
			11 PJF
			12 PJF
Paltamo (2008)	64°24′N	21.23	1KJF
	27°50′E		20KJF
			44 KJF
			49 KJF
Jyväskylä (2008)	62°14′N	19.53	5SOF
	25°44′E		11SOF
			6SOF
			7SOF
Lahti (2009)	60°59′N	19.02	L809
	25°39′E		L709
			L109
			L409

### Determination of CDL for reproductive diapause

*Drosophila montana* flies were collected from five cline populations (Pelkosenniemi 67°N; Oulanka, 66°N; Pudasjärvi, 65°N; Paltamo, 64°N, and Jyväskylä, 62°N) in autumn 2008. In spring–summer 2009, we determined the CDLs for *D. montana* females from these populations (four isofemale lines per locality) by studying the diapause incidence of females held in four light:dark (LD) cycles: 16:8, 17.5:6.5, 19:5, and 20.5:3.5. However, at the end of summer 2009, we managed to get flies from a sixth, more southern locality (Lahti, 60°N) and, to achieve a longer cline, we also determined CDLs for this population (four isofemale lines). In Lahti, the longest day in the summer lasts only about 19 h and so we omitted from this experiment LD 20.5:3.5 and added there two shorter day lengths 14:10 and 15:9. As the diapause percentages of females in Pudasjärvi, Paltamo, and Jyväskylä had not reached 100% in LD 16:8 in the first set of experiments, we studied the diapause responses of isofemale lines from these populations also in LD cycles 14:10 and 15:9.

The flies were sexed under light CO_2_ anesthesia within one day after their emergence before the sensitive period for diapause (Salminen T.S., unpubl. ms.) to obtain 80–150 females per line for each photoperiod. Females were put in vials (15 individuals/vial) containing 7 mL of yeast-sucrose-agar medium (Rosato and Kyriacou 2006) with some dry yeast on top and transferred to a climate chamber at 16°C (MEMMERT Model ICP 800; Memmert GmbH+Co.KG, Germany). Inside the climate chamber, separate wooden boxes were constructed, each with a different LD cycle and a light intensity of about 2.0×10^3^*l*x during the light phase. Females were maintained in these conditions for 21 days, that is, until they were sexually mature or had entered reproductive diapause. On day 22, they were transferred to –80°C and stored there until we determined their reproductive status by dissecting the ovaries under a light microscope. If the ovaries were small and transparent and/or if they contained only a little yolk and showed minimal structurization, the females were considered to be “diapausing.” If the ovaries were almost or completely developed and the ovarioles’ filaments were well formed and full of yolk, the females were classified as “reproducing” (“non-diapausing”). However, while this classification is straightforward in short and long day lengths, the ovaries show more variation in their shape, transparency, and nutrient accumulation when the day length approaches the CDL. As a result, near the CDL, our classification of females as “reproducing” was slightly relaxed to include those where at least one well-formed ovariole filament was found. For each locality of the cline, the CDL for female reproductive diapause was determined by calculating the percentages of diapausing females from four isofemale lines tested in each LD cycle.

To check how well our data fit the hypothesis of correlation between the locality/latitude and CDL, we used nonlinear regression analysis in the drc package in R (Team 2008; Ritz and Streibig 2005). With this software, we estimated CDLs for each isofemale line (data not shown) as well as mean CDLs for each population. We also checked for an association between latitude and CDL using linear regression analysis of CDL estimations against latitude where each isofemale line was considered as a separate datapoint. A Shapiro–Wilk test was used to test for deviations from normality, and a Bartlett test was performed to check equality of variances.

### DNA extraction and microsatellite genotyping

Genomic DNA was extracted from wild-caught individuals of both sexes (Table 2) using the Qiagen DNeasy Tissue Kit (QIAGEN, Germany) according to the manufacturer's protocol. Genetic differentiation/gene flow between populations was estimated using 12 polymorphic microsatellite markers: vir4, vir11, vir17, vir74 and vir93 (Routtu et al. 2007), mon1 and mon29 (Orsini et al. 2004), vir19 and vir71 (Huttunen and Schlötterer 2002), and vir13, vir103, and vir104 (Schäfer, pers. comm.). Polymerase chain reaction (PCR) conditions and reagent concentrations were according to Routtu et al. 2007. DNA fragments were denaturated in formamide, run on an ABI 3100 genetic analyzer, and analyzed with GeneMapper 3.7 software (both Applied Biosystems, USA).

**Table 2:** Sample size and heterozygosity estimates for the study populations

Cline populations	Population code	Number of individuals	*H*_O_ (SD)	*H*_E_ (SD)	Heterozygote deficiency test (*P*-values)
Pelkosenniemi	PT	29	0.726 (0.118)	0.782 (0.061)	0.032
Oulanka	OUL	38	0.712 (0.077)	0.756 (0.054)	0.132
Pudasjärvi	PJ	20	0.750 (0.090)	0.772 (0.051)	0.101
Paltamo	PA	28	0.734 (0.081)	0.754 (0.086)	0.094
Jyväskylä	JKL	14	0.715 (0.157)	0.815 (0.061)	0.000
Lahti	LA	9	0.593 (0.197)	0.767 (0.116)	0.000

### Population differentiation analysis

Population structure (*F*_ST_) of *D. montana* cline populations was estimated using data from the 12 microsatellite loci. To test genotypic linkage disequilibrium and Hardy–Weinberg equilibrium (HWE), we used GENEPOP 4.0.10 software package (Raymond and Rousset 1995) employing Bonferroni correction (Rice 1989) to the linkage disequilibrium results. Presence of null alleles was tested with MICRO-CHECKER 2.2.3 software (Van Oosterhout et al. 2004). Heterozygosity values were estimated with ARLEQUIN, version 3.5.1.2 (Excoffier et al. 2005) and *P*-values for heterozygote deficiency were estimated with Score (U) test in GENEPOP 4.0.10. To get another estimate of differentiation, we also calculated *D*_EST_ differentiation statistics (*D*_ST_, Jost 2008). Pairwise *F*_ST_ and *D*_ST_ estimate comparisons between pairs of populations were obtained with “DEMEtics” package in R (Team 2008; Gerlach et al. 2010). Finally, population structure was estimated with STRUCTURE 2.3.1 software (Pritchard et al. 2000), for which we used both admixture and no-admixture models assuming different numbers of *K* (from 1 to 7) with a burn-in period of 30,000 generations and 1,000,000 Markov Chain Monte Carlo (MCMC) iterations. Isolation by distance was tested using a Mantel test in GENEPOP version 4.0.10.

## Results

### CDL for reproductive diapause

**Figure 2:**
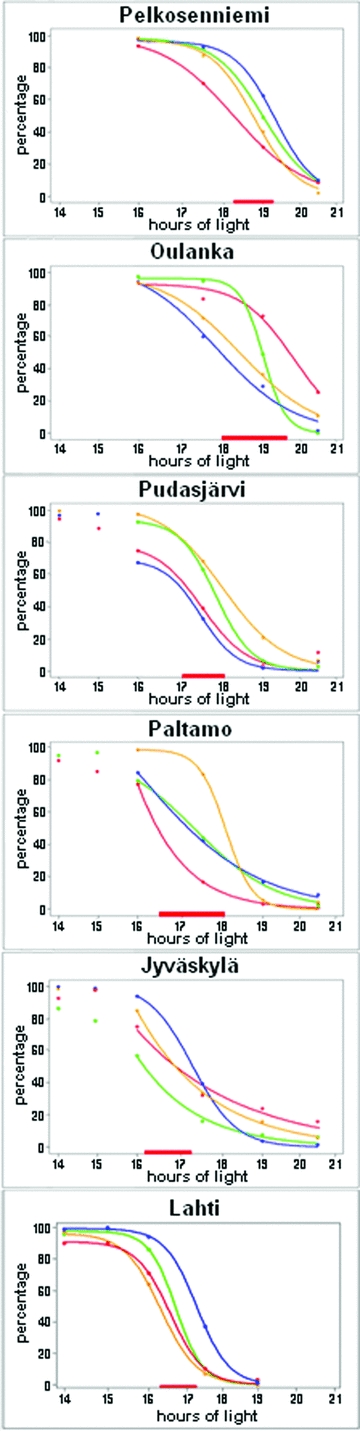
Proportion of diapausing females in four isofemale lines from different sampling localities in different LD cycles. Critical day length (CDL) is indicated with a thick line on the *x*-axes. The points not connected with the lines (on the left side of the graphs for Pudasjärvi, Paltamo, and Jyväskylä) represent the extra LD cycles used in the second set of the diapause experiment (see text for details).

**Figure 3:**
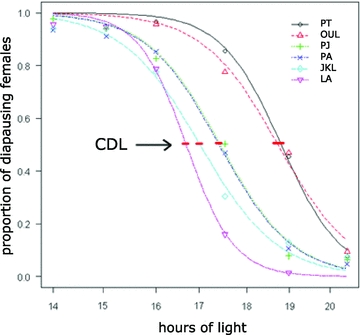
The graphical result of dose-response model showing correlation between latitude and average CDL for each population.

Our data showed a clear correlation between the CDL, at which 50% of females enter diapause, and the latitude from where the *D. montana* samples originated (Figs. 2 and 3). The CDLs of the most northern cline populations ranged between 18 and 19.6 h (Fig. 2), the estimated mean for Pelkosenniemi and Oulanka being about 18.8 h. CDLs of the more southern localities were clearly shorter: the estimated mean for Pudasjarvi and Paltamo was about 17.3 h and for Jyväskylä and Lahti, 16.6–16.9 h (Fig. 2; Table 3). The dose-response model showed that the CDLs of the cline populations differ from each other according to their location, *P*-values for all estimates were highly significant. The analysis was performed in two ways: using the data from the first part of the experiment for five populations and using all the data. There was no difference in the result. Also, regression analysis (latitude as a predictor, CDL as a response) revealed a significant relationship between these parameters (for five populations: *b*= 0.387, SE = 0.093, *R*^2^= 0.507, *P* < 0.001; for all the data: *b*= 0.333, SE = 0.058, *R*^2^= 0.615, *P* < 0.001). Variation in CDLs within populations was 1–1.7 h (Fig. 2).

**Table 3:** Average CDL for cline populations

Population	Average CDL for cline populations (SE)	Approximate date in the population corresponding to the mean CDL
Pelkosenniemi	18.8 (0.14)	3rd August
Oulanka	18.7 (0.16)	2nd August
Pudasjärvi	17.3 (0.15)	10th August
Paltamo	17.4 (0.15)	8th August
Jyväskylä	16.9 (0.15)	6th August
Lahti	16.6 (0.12)	9th August

All *P*-values are not significant.

### Genetic differentiation and population structure

Testing for population differentiation did not reveal any structure among the *D. montana* flies from different sampling sites. Four of the 12 loci (vir71, vir103, vir104, and mon29) showed strong deviations from HWE due to null alleles in all populations (data not shown) and were discarded from further analyses. Pairwise *F*_ST_ and *D*_ST_ comparison, calculated between pairs of cline populations, (Table 4), were quite low; overall *F*_ST_ was 0.0001 and *D*_EST_ estimator for all populations was 0.002. Population genetic estimates such as observed (*H*_0_) and expected (*H*_E_) heterozygosity values are given in Table 2. The significant heterozygote deficiency values in Jyväskylä and Lahti populations could be partly explained by sampling effect (low sample sizes), but deficiency in Pelkosenniemi may be due to additional unobserved null alleles. Clustering of multilocus genotypes with STRUCTURE software indicated a single population with no population structure, that is, random gene flow between sampling sites. Interestingly, despite the low population differentiation, the Mantel test revealed a significant correlation between geographic and genetic distances (*R*^2^= 0.464; *P*= 0.038) indicating limited gene flow between the most distant populations.

**Table 4:** Pairwise *F*_ST_ (lower semimatrix) and *D*_ST_ (upper semimatrix) differences

	Pelkosenniemi	Oulanka	Pudasjärvi	Paltamo	Jyväskylä	Lahti
Pelkosenniemi	-	−0.0099	0.0095	−0.0122	−0.0170	0.0749
Oulanka	−0.0012	-	0.0036	−0.0055	0.0227	0.0915
Pudasjärvi	0.0014	0.0004	-	−0.0060	−0.0098	0.0576
Paltamo	−0.0014	−0.0009	−0.0016	-	−0.0118	0.0564
Jyväskylä	−0.0008	0.0038	−0.0016	−0.0006	-	0.0350
Lahti	0.0066	0.0082	0.0039	0.0046	0.0017	-

None of the outlined values are statistically significant.

## Discussion

Insects living at high latitudes face the demanding task of having to adjust the most important stages of their life cycle to match seasonal changes in their environment, and to do this, the majority of insects rely on photoperiodic cues (Tauber and Tauber 1986). In our study, the difference in the average CDLs between the northernmost and southernmost ends of the *D. montana* cline was approximately 2 h. This corresponds well with Danilevsky's rule that the CDL in genetic clines lengthens 1– 1 h and 30 min per 5° of latitude northwards (Danilevsky, Goryshin and Tyshchen 1970). Moreover, the CDLs of the six cline populations strongly correlated with latitude even though the distances between the sampling sites were relatively short (110–315 km). Variation in CDLs matches approximately the same time of summer in all study populations. However, the “time window” for diapause induction in the northernmost cline population is much narrower than that in the more southern locations. In Pelkosenniemi (approximately 67°N), shortening the day length by 1 h takes about 1 week in July/August, while in Lahti (approximately 61°N), a change of the same magnitude requires almost 2 weeks.

Two other factors, temperature and nutrition, can affect the onset and maintenance of diapause along with the photoperiod. While photoperiod is the most reliable environmental cue for detecting the closeness of approaching winter in the northern latitudes, in the south, the changes in day length with latitude are less extreme and the role of temperature becomes more important. According to Charlesworth and Shorrocks (1980), photoperiodic diapause induction operates in *Drosophila* species only within a rather narrow range of temperatures, which varies between the species. For example, in many cosmopolitan species, such as *D. melanogaster*, the females enter diapause in short day length only when the temperature is below 14°C (Saunders and Gilbert 1990). Also in *D. littoralis*, which belongs to the same species group as *D. montana*, the percentage of diapausing females is much lower in 22°C than in 16°C (Lumme et al. 1974). While the temperature is not known to evoke diapause per se, that is without changes in photoperiodic cues, in any Drosophila species, high temperatures can postpone the onset of diapause at the population level and adjust the CDLs toward longer day lengths (e.g., Ichijo 1986; Pittendrigh and Takamura 1987). This is due to phenotypic plasticity in females’ responses to photoperiodic cues, which allows the females to be more flexible in the timing of diapause if the warm period varies between the years. Temperature also affects the termination of diapause in spring inducing variation in the number of generations per year (voltinism) between latitudes.

The average day lengths corresponding to the CDLs of *D. montana* cline populations in Finland fall in the beginning of August, suggesting that nearly all females emerging during August in these populations will enter diapause. In the northern parts of the cline, late spring allows the populations to have only one generation per year, since the progenies of overwintered flies emerge only after the CDL in August. For example, in Oulanka (66°N), the flies of the *D. virilis* group species, including *D. montana*, have a gap between the adult generations in July, when the overwintered flies have already died and the first summer generation has not yet emerged (e.g., Lumme 1978; Lumme et al. 1979). However, in the southern parts of the cline, earlier spring combined with a longer warm period allows the populations to be partly bivoltine. Already in Kemi (65°N; not included in this study), *D. montana* has a partial second generation in July (Aspi et al. 1993). Even though Kemi is only 1° latitude south from Oulanka, it is close to sea and the flies are active about 1 month earlier than in Oulanka. The most southern population of *D. montana* has been found in Colorado, USA, at 39°N, but it is located at an altitude of nearly 3000 m (Baker 1975), where the winter is as long and cold as in Oulanka. The daily changes in day length at this latitude are very small (only 0.3 hours per week in July/August) and so the females may need to track temperature changes in addition to changes in photoperiod to enter diapause at the right time. All females collected in August from Colorado have been found to be diapausing (Baker 1975).

In our study, the four *D. montana* isofemale lines per location showed approximately 1 –1.7 h variation in CDLs within the cline populations. P. Lankinen (unpubl. ms.) has found that the CDLs of 14 *D. montana* isofemale lines from one of our study populations (Oulanka) vary by about 2 h, which suggests that our data using four lines per location already give a fairly good estimate of the amount of within population variation. All of our study strains had been collected in autumn and kept in the laboratory under diapause-preventing conditions for about 6 months before using the flies in the experiments. In these conditions (constant light, 19°C), the diapause behavior of the flies remains invariable for years (Oikarinen and Lumme 1979). Also, the fact that the study was performed in two parts did not affect the results. The mean CDLs of the study populations as well as the correlation between the CDLs and latitudes were of the same magnitude for the data collected during the first year as for the whole dataset. Local variation in the quantity and quality of genetic variation in the CDLs could be estimated more effectively with a selection experiment, which we plan to perform in future.

The wide variation in CDLs within the cline populations detected in our study may be partly caused by migration of flies adapted to more southern or northern conditions, as our study also revealed no restrictions to gene flow between the cline populations. On the other hand, relatively low dispersal ability of the flies supports our finding that the cline populations farthest apart are isolated by distance. In nature, *D. montana* flies can migrate with average dispersal rate of about 1 km/day depending on environmental conditions, such as food access, humidity, and wind velocity, among others (Aspi et al. 1993). Variation in CDLs within the cline populations could also be maintained by alternating selection pressures within the populations. In some years, it may be beneficial for the females emerging in late summer to produce progeny instead of entering diapause, while in other years this may be a detrimental strategy. Also, diapause may have different kinds of trade-offs and correlations with other life-history traits in wild populations, and in bivoltine populations, selection may favor different life-history traits in summer and in overwintering generations, which may further complicate the situation.

The finding that local adaptation can lead to strong latitudinal clines in phenotypic characters in spite of gene flow is not unique. Demont et al. (2008) found variation in five life-history traits (including diapause induction and diapause duration) in 10 populations of yellow dung fly along a latitudinal cline across Europe. Also, Sarup et al. (2009) studied five populations of *D. buzzatii* and *D. simulans* along an altitudinal cline in La Gomera (Canary Islands) finding patterns of local adaptation in stress-related traits. Both of these studies included microsatellite analyses and showed that gene flow between populations was high. However, the fact that microsatellite markers do not indicate any population structure does not rule out the possibility that the cline populations could differ from each other at specific chrosomosomal areas or genes that are under selection. Another, more powerful, approach for tracing the differences between the cline populations would be to perform genome scans for the most northern and southern populations of the cline. This method might also help identify “genomic islands,” that is those parts of genome that have diverged between populations due to different selection pressures, or to detect “regions” of local adaptation at the genetic level (e.g., Wood et al. 2008). It would also be interesting to trace variation in the structure and splicing of specific candidate genes known to affect diapause and/or traits connected with it along the latitudinal cline. Such studies may eventually lead to a better understanding of the genetic architecture of diapause and help determine how genetic variation for this important life-history trait is maintained in natural populations.

## References

[b1] Aspi J, Lumme J, Hoikkala A, Heikkinen E (1993). Reproductive ecology of the boreal riparian guild of Drosophila. Ecography.

[b2] Baker WK (1975). Linkage disequilibrium over space and time in natural populations of *Drosophila montana*. Proc. Nat. Acad. Sci..

[b3] Bale JS, Hayward SA (2010). Insect overwintering in a changing climate. J. Exp. Biol..

[b4] Bradshaw W (1976). Geography of photoperiodic response in diapausing mosquito. Nature.

[b5] Charlesworth P, Shorrocks B (1980). The reproductive biology and diapause of the British fungal-breeding Drosophila. Ecol. Entomol..

[b6] Danilevskii AS (1965). Photoperiodism and seasonal development of insects.

[b7] Danilevsky AS, Goryshin NI, Tyshchen VP (1970). Biological rhythms in terrestrial arthropods. Annu. Rev. Entomol..

[b8] Danks HV (1987). Insect dormancy: an ecological perspective.

[b9] Demont M, Blanckenhorn WU, Hosken DJ, Garner TWJ (2008). Molecular and quantitative genetic differentiation across Europe in yellow dung flies. J. Evol. Biol..

[b10] Denlinger DL (2002). Regulation of diapause. Ann. Rev. Entomol..

[b11] Endler JA (1977). Geographic variation, speciation and clines.

[b12] Excoffier L, Laval G, Schneider S (2005). Arlequin (version 3.0): an integrated software package for population genetics data analysis. Evol. Bioinform. online.

[b13] Gerlach G, Jueterbock A, Kraemer P, Deppermann J, Harmand P (2010). Calculations of population differentiation based on GST and D: forget GST but not all of statistics!. Mol. Ecol..

[b14] Hahn DA, Denlinger DL (2007). Meeting the energetic demands of insect diapause: nutrient storage and utilization. J. Insect Physiol..

[b15] Huttunen S, Schlötterer C (2002). Isolation and characterization of microsatellites in *Drosophila virilis* and their cross species amplification in members of the *D. virilis* group. Mol. Ecol. Notes.

[b16] Ichijo N (1986). Disjunctive cline of critical photoperiod in the reproductive diapause of *Drosophila lacertosa*. Evolution.

[b17] Jost L (2008). GST and its relatives do not measure differentiation. Mol. Ecol..

[b18] Lakovaara S (1969). Malt as a culture medium for Drosophila species. Drosoph. Inf. Serv..

[b19] Lankinen P (1986). Geographical variation in circadian eclosion rhythm and photoperiodic adult diapause in *Drosophila littoralis*.

[b20] Lumme J, Dingle H (1978). Phenology and photoperiodic diapause in northern populations of Drosophila. Evolution of insects migration and diapause.

[b21] Lumme J, Lakovaara S, Muona O, Jarvinen O (1979). Structure of a boreal community of drosophilids (Diptera). Aquilo Ser. Zool..

[b22] Lumme J, Oikarinen A, Lakovaara S, Alatalo R (1974). The environmental regulation of adult diapause in *Drosophila littoralis*. J. Insect Physiol..

[b23] Musolin DL, Numata H (2003). Timing of diapause induction and its life-history consequences in Nezara viridula: is it costly to expand the distribution range?. Ecol. Entomol..

[b24] Oikarinen A, Lumme J (1979). Selection against photoperiodic reproductive diapause in *Drosophila littoralis*. Hereditas.

[b25] Orsini L, Huttunen S, Schlotterer C (2004). A multilocus microsatellite phylogeny of the *Drosophila virilis* group. Heredity.

[b26] Pittendrigh CS, Takamura T (1987). Temperature dependence and evolutionary adjustment of critical night length in insect photoperiodism. Proc. Nat. Acad. Sci..

[b27] Pritchard JK, Stephens M, Donnelly P (2000). Inference of population structure using multilocus genotype data.. Genetics.

[b28] Raymond M, Rousset F (1995). GENEPOP (Version 1.2): population genetics software for exact tests and ecumenicism. J. Hered..

[b29] Rice WR (1989). Analyzing tables of statistical tests. Evolution.

[b30] Ritz C, Streibig JC (2005). Bioassay analysis using R. J. Stat. Softw..

[b31] Rosato E, Kyriacou CP (2006). Analysis of locomotor activity rhythms in Drosophila. Nat. Protocols.

[b32] Routtu J, Hoikkala A, Kankare M (2007). Microsatellite-based species identification method for *Drosophila virilis* group species. Hereditas.

[b33] Sarup P, Frydenberg J, Loeschcke V (2009). Local adaptation of stress related traits in *Drosophila buzzatii* and *Drosophila simulans* in spite of high gene flow. J. Evol. Biol..

[b34] Saunders DS, Gilbert LI (1990). Regulation of ovarian diapause in *Drosophila melanogaster* by photoperiod and moderately low temperature. J. Insect Physiol..

[b35] Schluter D (2001). Ecology and the origin of species. Trends Ecol. Evol..

[b36] Schmidt PS, Paaby AB (2008). Reproductive Diapause and life-history clines in North American populations of *Drosophila melanogaster*. Evolution.

[b37] Tauber E, Kyriacou BP (2001). Insect photoperiodism and circadian clocks: models and mechanisms. J. Biol. Rhythms.

[b38] Tauber MJ, Tauber CA (1986). Seasonal adaptations of insects.

[b39] Team RDC (2008). R: a language and environment for statistical computing.

[b40] Throckmorton LH, Ashburner M, Carson HL, Thompson JN (1982). The virilis species group. The genetics and biology of drosophila..

[b41] Van Oosterhout C, Hutchinson WF, Wills DPM, Shipley P (2004). Micro-Checker: software for identifying and correcting genotyping errors in microsatellite data.. Mol. Ecol. Notes.

[b42] Via S (2009). Natural selection in action during speciation. Proc. Nat. Acad. Sci. U.S.A..

[b43] Wood HM, Grahame JW, Humphray S, Rogers J, Butlin RK (2008). Sequence differentiation in regions identified by a genome scan for local adaptation. Mol. Ecol..

